# Pharmacological, surgical and diagnostic innovations in Meniere’s disease: a review

**DOI:** 10.37825/2239-9747.1009

**Published:** 2020-10-01

**Authors:** A Scarpa, M Ralli, E De Bonis, D Troisi, A Montanino, P Viola, G Chiarella, FM Gioacchini, M Cavaliere, E Cassandro, C Cassandro

**Affiliations:** 1 Department of Medicine and Surgery, University of Salerno, Salerno, Italy; 2 Department of Sense Organs, Sapienza University Rome, Rome, Italy; Center for Hearing and Deafness, University at Buffalo, Buffalo, NY 14214, USA; 3 Department of Experimental and Clinical Medicine, Unit of Audiology, Regional Centre for Cochlear Implants and ENT Diseases, Magna Graecia University, Catanzaro, Italy; 4 Ear, Nose, and Throat Unit, Department of Clinical and Molecular Sciences, Polytechnic University of Marche, Ancona, Italy; 5 Surgical Sciences Department, University of Turin, Turin, Italy

**Keywords:** Meniere’s disease, endolymphatic hydrops, glycerol, intratympanic therapy, diuretics, gentamicin

## Abstract

Meniere’s disease is an inner ear disorder characterized by the presence of endolymphatic hydrops in the inner ear and symptomatology of recurrent and debilitating vertigo attacks, tinnitus, aural fullness, and fluctuating sensorineural hearing loss. Although many therapeutic options for MD have been proposed during years, no consensus has been reached by the scientific community. In the last decade, many therapeutic options have been proposed, as intratympanic steroid, intratympanic gentamicin, and intravenous glycerol. Recently, the role of the antisecretory factor in the diet of MD patients have been investigated. Surgery is recommended for intractable MD; some authors proposed new approaches including transcanal endoscopic infracochlear vestibular neurectomy, new marsupiliazation technique in sac surgery, and tenotomy of the stapedius and tensor tympani muscles.

## I. INTRODUCTION

Meniere disease (MD) is an inner ear disorder characterized by the presence of endolymphatic hydrops (EH) in the inner ear and symptomatology of recurrent and debilitating vertigo attacks, tinnitus, aural fullness, and fluctuating sensorineural hearing loss (SNHL)^1,2,3^. Although hearing loss is mainly sensorineural, low-frequency air-bone gap (ABG) can also be found in the absence of middle ear pathology^4^, mimicking other conditions such as cerebral vascular anomalies including dural arteriovenous fistula^5^. MD symptoms can severely affect the quality of life^6,7^.

Although many therapeutic options for MD have been proposed, no consensus has been reached by the scientific community^8–11^. First-line treatment includes dietary restrictions for salt, caffeine, and alcohol associated to drugs both for acute attacks (dimenhydrinate, benzodiazepines)^12^, and as prophylactic therapy (betahistine, β-blockers, diuretics)^13^.

When first-line treatment does not offer satisfactory symptom control, intratympanic (IT) administration of gentamicin or corticosteroids can be performed^14^; however, many studies showed that IT gentamicin may expose the patients to a risk of hearing loss, depending on dosage and intervals between administrations^15^. Labyrinthectomy or other surgical procedures can be suggested for intractable MD^16^.

Despite the recent COVID-19 pandemic^17–20^, scientific research for MD is a growing area in continuous development. This brief review aims to assess the clinical innovations for the therapy of MD.

## II. DISCUSSION

### Pharmacological Innovations

The main goal of the pharmacological treatment of MD is to reduce the duration and frequency of vertigo attacks, and to prevent hearing loss, which is usually progressive. There’s lack of high-level evidences for efficacy of MD treatment; for this reason, different drugs and different methods of administration have being tested.

IT steroids (dexamethasone, methylprednisolone) treatments have been widely used for MD, especially when first-line treatment does not offer a satisfactory symptoms control; however, there is still lack of evidence that this treatment may have a positive effect in MD^21^. Pradhan et al^22^ evaluated the effects of IT dexamethasone in 30 patients with refractory MD comparing the pre- and post-treatment hearing outcome and dizziness score. They found that 23% of patients were free of vertigo at the end of 24 months after IT steroids, but there was no significant improvement in hearing. These results are in accordance with those from Weckel et al^23^ who retrospectively evaluated 25 patients treated with IT dexamethasone; they reported a satisfactory control of vertigo in 92% of the patients, and this protocol achieved control of vertigo in 70% of patients at 2 years. Harcourt et al^24^ suggested IT methylprednisolone as an effective treatment for the long-term control of vertigo attacks, without he known inner-ear toxicity associated with gentamicin.

Gentamicin, administered at different doses and timing^25^, has been proven as an effective treatment for vertigo control in MD with a potential risk of herring loss; however, discussion about the dosage and the method used is open^26^. Scarpa et al^27^ proposed a low-dose IT gentamicin protocol; they treated 48 patients with 0.5 ml of 10 mg of gentamicin with an interval of 2 weeks between injections. They reported a satisfactory control of vertigo attacks after IT gentamicin, and the effect of this protocol on vestibular system was proved by the reduction in vestibulo-ocular reflex (VOR) gain in the affected side. A meta-analysis from Jian et al^28^ demonstrated that IT gentamicin is superior to IT steroids in reducing the number of vertigo attacks, but both drugs didn’t show hearing improvement. Moreover, Ozturk and Ata proposed an IT mixture of gentamicin and dexamethasone injection for the treatment of intractable MD, even more effective that IT dexamethasone for vertigo control^29^.

Diuretics have also been recommended to control vertigo attacks in MD, due to their effects in reducing the endolymphatic pressure and volume. As for steroids and gentamicin, there are no high-quality data demonstrating the efficacy of oral diuretic therapy^30^; in addition, a meta-analysis from Rosenbaum and Winter^31^ concluded that it is not clear if diuretic lead to a symptomatic improvement of vertigo.

A prospective study from Scarpa et al^32^ evaluated the effectiveness on vertigo control of intravenous (IV) glycerol (an osmotic diuretic), 10% glycerol with 0.9% sodium chloride, 0.5 g/kg once a day for 2 consecutive days every fifteen days for six months; the authors reported an improvement of vertigo attacks and a significant reduction of discomfort generated by tinnitus, raising quality of life.

Recently, some authors suggested a possible action of anti-secretory factor (AF), a protein produced by pituitary gland that plays a role in the innate defense against the inflammatory and secretory components of diarrheal disease^33^. It has been hypothesized that AF can act as a modulator of water and ions and interact with the aquaporins. Viola et al^34^ observed a significant improvement in daily activities in patients treated with specially processed cereals (SPC) compared to those treated with IV glycerol and dexamethasone. The study reported a significant reduction of vertigo spells and a positive effect on tinnitus severity.

#### Surgical Innovations

According to international guidelines, surgical therapy for MD is recommended only for refractory disease, and therefore represents the third (or even the fifth) line of management. Currently, the most popular surgical procedures to control vertigo attacks in MD are also the most aggressive and those that have the most negative impact on auditory function. Besides, there is a lack of evidence suggesting that surgical therapy for MD may provide a significant control of symptoms^35^.

The table below shows the main surgical procedures for MD taking into account of hearing preservation, vestibular injury, surgical difficulty and long-term outcomes (Table 1).

**Table:** 

Procedures	Hearing preservation	Vestibular injury	Surgical difficulty	Long-term outcomes
Endolymphatie sac surgery	Yes	No	Medium	Poor
Grommet insertion	Yes	No	Easy	Poor
Tenotomy of ear muscles	Yes	No	Easy	Few studies
Transmastoid labyrinthectomy	No	Yes	High	Good
Vestibular neurectomy	Yes	No	High	Good
Triple semicircular canal plugging	Yes	Yes	High	Good

Endolymphatic sac surgery (ESS) can be considered in patients that are refractory to medical management as a nondestructive option, as the risk to damage hearing is low^36^. Xu et al^37^ suggested that ESS with posterior tympanotomy and local steroid treatment could improve hearing and ensure a satisfactory vertigo control in patients with intractable MD. In Gibson et al opinion^38^, ESS can provide a vertigo control at least as well as IT gentamicin with a lower incidence of audio-vestibular complications^39^. Transmastoid labyrinthectomy alone^40^ or in combination with cochlear implantation^41^, grommet insertion^42^, and triple semicircular canal plugging^43^ have been proposed as an effective surgical option for the treatment of MD.

New techniques have been recently described. Daneshi et al^44^ evaluated the results of a new marsupialization technique in endolymphatic sac decompression surgery, in which the outer layer of the sac was turned around and placed under the anterior bony border. They concluded that this technique can control progressive hearing loss and improve tinnitus, vertigo, and ear fullness among patients with intractable unilateral MD.

Moreover, Trakimas et al^45^ proposed a transcanal endoscopic infracochlear vestibular neurectomy on cadaver as a minimally invasive approach to distal vestibular neurectomy.

More recently, some authors suggested that tenotomy of the stapedius and tensor tympani muscles (TSTM) may be a safe surgical procedure with significant vertigo control, decreased postoperative symptoms and important hearing preservation^46^.

#### Diagnostic Innovations

In the diagnosis and the evaluation of therapy efficacy, the audio-vestibular test battery has a primary role, and the diagnostic findings are constantly evolving.

Di Stadio et al proposed head-shaking nystagmus (HSNy) as a useful test in the early stages of MD to predict a new vertigo attack, with a sensitivity of the ipsilesional HSNy of 100% at T0 and 85.7% at T1^47^.

Lee SU et al^48^ evaluated the VOR performance during the attacks of MD using video head-impulse tests (video-HITs) according to each ictal phase; the authros suggested that during the vertigo attack, HITs are normal during irritative and recovery phases, but abnormal in 53% of the patients during the paretic phase, reflecting characteristic ictal vestibular discharges. Besides, a common finding in MD is a normal horizontal HIT with an asymmetric caloric function, and this dissociation can be used as a diagnostic marker^49^.

Patients with suspected MD can be examined using magnetic resonance imaging (MRI) to evaluate a possible inner ear disease. The MRI findings in patients with MD are conflicting, due to the MRI sequences used and the inclusion criteria of the patients; also, the early stage of MD and the early symptoms appear too subtle for identification using MRI, making the reproducibility of hydrops MRI scan protocols debatable^50^.

## III. CONCLUSION

Despite accumulating evidence for the treatment of MD, there is currently no international consensus on a standardized therapeutic protocol for this condition. Intravenous administration of glycerol, low-dose intratympanic gentamicin, and antisecretory factor are promising innovations.

The role of surgery in the treatment of MD is still debated; recent techniques as tenotomy of the stapedius and tensor tympani muscles seems to offer interesting insights, but larger randomized studies are needed to draw conclusions.
